# Cardiac Ankyrin Repeat Protein Attenuates Cardiac Hypertrophy by Inhibition of ERK1/2 and TGF-β Signaling Pathways

**DOI:** 10.1371/journal.pone.0050436

**Published:** 2012-12-05

**Authors:** Yao Song, Jialin Xu, Yanfeng Li, Chunshi Jia, Xiaowei Ma, Lei Zhang, Xiaojie Xie, Yong Zhang, Xiang Gao, Youyi Zhang, Dahai Zhu

**Affiliations:** 1 Department of Biochemistry and Molecular Biology, Institute of Basic Medical Sciences, Chinese Academy of Medical Sciences and Peking Union Medical College, Beijing, China; 2 Institute of Vascular Medicine, Peking University Third Hospital, Key Laboratory of Cardiovascular Molecular Biology and Regulatory peptides, Ministry of Health and Key Laboratory of Molecular Cardiovascular Science, Ministry of Education, Beijing, China; 3 National Resource Center for Mutant Mice Model Animal Research of Nanjing University, Pukou High-Tech District, Nanjing, China; 4 Department of Cardiology, Second Affiliated Hospital, Zhejiang University College of Medicine, Hangzhou, China; University of Torino, Italy

## Abstract

**Aims:**

It has been reported that cardiac ankyrin repeat protein is associated with heart development and diseases. This study is aimed to investigate the role of CARP in heart hypertrophy in vivo.

**Methods and Results:**

We generated a cardiac-specific CARP-overexpressing transgenic mouse. Although such animals did not display any overt physiological abnormality, they developed less cardiac hypertrophy in response to pressure overload than did wildtype mice, as indicated by heart weight/body weight ratios, echocardiographic and histological analyses, and expression of hypertrophic markers. These mice also exhibited less cardiac hypertrophy after infusion of isoproterenol. To gain a molecular insight into how CARP attenuated heart hypertrophy, we examined expression of the mitogen-activated protein kinase cascade and found that the concentrations of phosphorylated ERK1/2 and MEK were markedly reduced in the hearts of transgenic mice subjected to pressure overload. In addition, the expressions of TGF-β and phosphorylated Smad3 were significantly downregulated in the hearts of CARP Tg mice in response to pressure overload. Furthermore, addition of human TGF-β1 could reverse the inhibitory effect of CARP on the hypertrophic response induced by phenylephrine in cardiomyocytes. It was also evidenced that the inhibitory effect of CARP on cardiac hypertrophy was not attributed to apoptosis.

**Conclusion:**

CARP attenuates cardiac hypertrophy, in which the ERK and TGF-β pathways may be involved. Our findings highlight the significance of CARP as an anti-hypertrophic factor in therapy of cardiac hypertrophy.

## Introduction

Cardiac hypertrophy is an adaptive response of the myocardium to the increased workload that results from diverse cardiovascular diseases. Although this compensatory response to stress is considered to be an effective means to support increased cardiac output, prolonged hypertrophy ultimately leads to sudden death or progression to heart failure [1]. Pathological stress signals usually initiate cardiac hypertrophy through two classes of mechanisms: biomechanical/stretch-sensitive mechanisms and neurohumoral mechanisms [2]. Whichever mechanism serves as the initiating stimulus, the hypertrophic response is switched on at the level of receptors or ion channels, which activate intracellular signaling cascades and transcriptional factors. The ultimate result is cardiomyocyte hypertrophy, fibroblast hyperplasia, and activation of the “fetal gene” program. An imbalance between the expression of pro- and anti-hypertrophic factors acting via a network of intracellular signaling pathways is responsible for development of cardiac hypertrophy [3]. However, previous research efforts have focused largely on signaling pathways that positively regulate cardiac hypertrophy. By comparison, negative regulators of cardiac hypertrophy have received much less attention. Accordingly, the therapeutic measures against cardiac hypertrophy developed to date principally target pro-hypertrophic pathways; however, patient outcomes are far from ideal [4]. Against this backdrop, the development of new therapies aimed at enhancing the anti-hypertrophic effect is arguably a worthy undertaking.

CARP (cardiac ankyrin repeat protein), encoded by the *Ankrd1* (ankyrin repeat domain 1) gene, was originally identified in human dermal microvascular endothelial cells induced with interleukin (IL)-1A and tumor necrosis factor α (TNFα) [4], and was subsequently shown to be expressed predominantly in the heart. Developmental studies showed that *Ankrd1* transcripts are first detected at 8.5 days post-coitus in mouse embryos; thereafter, *Ankrd1* continues to be abundantly expressed in the embryonic heart but levels decrease in the adult heart. This pattern of expression suggested that CARP might function to negatively regulate transcription of cardiac genes in the fetal heart [5]. Additional studies have implicated CARP in myofibrillar assembly, stretch sensing, and communication between the sarcoplasm and the nucleus in the adult heart [6], [7], [8].

The most intriguing clue to the possible functional role of CARP comes from the observation that expression of the *Ankrd1* gene is rapidly induced in response to various hypertrophic stimuli, including pressure overload, denervation, stretch, and neurohumoral agonists (e.g., phenylephrine, endothelin, angiotensin II, and isoproterenol) [9]. Recent studies have also indicated that the *Ankrd1* gene is strongly upregulated in the hearts of both hypertrophic animal models [10], [11], [12] and those of heart-failure patients with dilated cardiomyopathy (DCM), ischemic cardiomyopathy (ICM), or arrhythmogenic right ventricular cardiomyopathy (ARVC) [13], [14], [15]. These lines of evidence point to an important role for the CARP protein in heart development generally, and in cardiac hypertrophy in particular. Interestingly, however, mice with complete germline ablation of the *Ankrd1* gene do not show any phenotypic change during development. It is therefore necessary to establish animal models with heart-specific *Ankrd1* deletion and/or overexpression of CARP to further investigate the *in vivo* function of CARP during heart development and cardiac hypertrophy.

In the present study, we generated cardiac-specific CARP-overexpressing transgenic (CARP Tg) mice and used these animals as a hypertrophic model to investigate the functional role of CARP in cardiac hypertrophy. Our results show that CARP has an important role in inhibiting cardiac hypertrophy induced by pressure overload and continuous isoproterenol infusion, and reveal an important regulatory role for transforming growth factor-β (TGF-β) signaling and the mitogen-activated protein kinase (MAPK) cascade, specifically the MEK/ERK1/2 (MAPK/ERK kinase/extracellular signal-regulated kinase) pathway, in mediating attenuation of cardiac hypertrophy and fibrosis by CARP.

## Methods

All of the animal procedures were conducted in accordance with the Guide for the Care and Use of Laboratory Animals published by the US National Institutes of Health (NIH Publication No. 85-23, revised 1996) and were approved by the Institutional Animal Care and Use Committee of Chinese Academy of Medical Sciences & Peking Union Medical College.

### Generation of Transgenic Mice

Transgenic mice were produced by microinjection of the α-MHC-CARP construct into fertilized mouse embryos (129 strain background). Four independent transgenic lines (B, D, E, and F) were established. The F-line transgenic mouse was backcrossed with C57/BL6J animals for more than five generations to create a >98.5% C57/BL6J background. Full details are provided in Supporting Information S1.

### Animal Models

Pressure overload-induced cardiac hypertrophy was produced by transverse aortic constriction (TAC) surgery [16]. Male CARP Tg mice (9–11-weeks old) and wild-type (WT) littermates were anesthetized with ketamine/xylazine/atropine (100 mg/10 mg/1.2 mg per 1 kg IP). After the mice were confirmed in an anaesthetized state (e.g. no response to toe pinching), they were next ventilated by tracheal intubation using a rodent ventilator (Alcbio Corporation, Shanghai, China) with a tidal volume of 0.2 mL and a respiratory rate of 88 breaths/min. The chest was opened at the suprasternal fossa along the midsternal line, and the thymus glands were superiorly reflected. The transverse thoracic aorta between the innominate artery and the left common carotid artery was dissected, and a 6-0 silk suture was tied around the aorta against a 26-gauge needle. Both control groups underwent a sham operation involving thoracotomy and aortic dissection without constriction of the aorta. After 4 weeks, the mice were sacrificed by cervical dislocation after deep anesthesia with 2% isoflurane (Baxter Healthcare Corporation, New Providence, NJ, USA) and the ratios of heart weight to body weight (HW/BW) and tibia length (HW/TL) were determined.

The isoproterenol-induced cardiac hypertrophic model was established by infusing isoproterenol (Sigma, St Louis, Mo) in vivo using subcutaneously implanted micro-osmotic pumps (Alzet DURECT, Cupertino, CA; model 1002). In brief, 9–11-week-old male CARP Tg and WT mice were anesthetized with ketamine/xylazine/atropine (100 mg/10 mg/1.2 mg per 1 kg IP). After confirming an anaesthetized state of mice (e.g. no response to toe pinching), micro-osmotic pumps were implanted in the back of mice, and isoproterenol was delivered continuously at a rate of 30 mg/kg/day. The control mice received vehicle (100 µmol/L ascorbic acid). After 14 days, the mice were sacrificed by cervical dislocation after deep anesthesia with 2% isoflurane and the ratios of heart weight to body weight (HW/BW) and tibia length (HW/TL) were determined.

Cardiac hypertrophy and function were assessed by echocardiography before and after TAC surgery or isoproterenol infusion using a Vevo 770TM Imaging System (VisualSonics Inc., Toronto, Canada) equipped with a 30-MHz microprobe under anesthesia with 1.5% isoflurane allowing spontaneous breathing.

### Histological Analysis

Histological analysis of tissues was performed according to standard protocols [17]. Full details are provided in Supporting Information S1.

### Isolation and Culture of Rat Cardiomyocytes

Cardiomyocytes were isolated and cultured from 1–2-day-old neonatal Sprague-Dawley (SD) rats as described previously [18]. Briefly, a central thoracotomy was performed after the neonatal rats were deeply anaesthetized with 1.0% isoflurane. The hearts were quickly excised and immediately embedded in freezing hanks solution. Cardiomyocytes were dispersed by digestion with 0.1% (w/v) trypsin and 0.03% (w/v) collagenase at 37°C, then were collected after differential adhesion of non-cardiomyocytes and plated at a density of 150–200 cells/mm^2^. Cultures were maintained in DMEM supplemented with 10% (v/v) fetal bovine serum; 100 µmol/L bromodeoxyuridine was included to prevent fibroblast proliferation.

### Generation of Recombinant CARP-GFP Adenovirus

Full-length rat CARP cDNA with a C-terminal Myc tag was subcloned into the pAdTrack vector. The resulting plasmid was linearized by digestion with *Pme*I and was transformed into *Escherichia coli* BJ5183 cells together with the adenoviral backbone plasmid pAdEasy-1. Kanamycin-resistant recombinants were selected and recombination was confirmed by digestion with *Pac*I. The recombinant adenoviral plasmid was transfected into 293A cells to generate infectious CARP-GFP-expressing viral particles (Ad-CARP-GFP). Adenovirus was purified via standard CsCl ultracentrifugation and desalting procedures. Viral titers were determined by plaque assay.

### Immunocytochemistry and Cell Size Measurements

Immunocytochemistry was performed with cardiomyocytes as described in Supporting Information S1.

### Quantitative Real-time Reverse Transcription-polymerase Chain Reaction (RT-PCR)

Total RNA was extracted from ventricular tissue and cultured neonatal rat cardiomyocytes using TRIzol (Invitrogen, Carlsbad, CA). cDNA was synthesized from total RNA using AMV reverse transcriptase and oligo (dT) primers (Promega, Madison, WI), as instructed by the manufacturer. PCR-amplified target mRNAs were quantified using SYBR Green PCR Master Mix (Bio-Rad, Hercules, CA) and normalized to glyceraldehyde phosphate dehydrogenase (GAPDH) mRNA levels. The primer sequences for PCR were presented in Supporting Information S1.

### Quantification of TGF-β1 Protein Production

The concentrations of TGF-β1 in heart homogenates and supernatants of cultured neonatal rat cardiomyocytes were measured using a specific ELISA kit (Shanghai ExCell Biology, Inc., Shanghai, China) employing the double-antibody sandwich method according to manufacturer’s instruction. Full details are provided in Supporting Information S1.

### Nuclear Staining and Laser Confocal Microscopy

Cardiomyocytes were fixed with 4% paraformaldehyde and permeabilized in 0.2% Triton X-100 for 30 min at 37°C. The cells were sequentially stained for 15 min with 0.1 µg/ml Hochest 33342. Nuclear structure was visualized on a Leica laser confocal microscope.

### Western Blotting

Immunoblotting was performed with heart homogenates and cardiomyocytes as described in Supporting Information S1.

### Statistical Analysis

Data are expressed as means ± SEMs. Differences between two groups were analyzed using unpaired Student’s t-tests. Comparisons among different mice genotypes subjected to different treatments were made by two-way ANOVA. A *P*-value less than 0.05 was considered significant.

## Results

### Overexpression of CARP Inhibits Neurohumoral Agonist-induced Cardiomyocyte Hypertrophy *in vitro*

**Figure 1:**
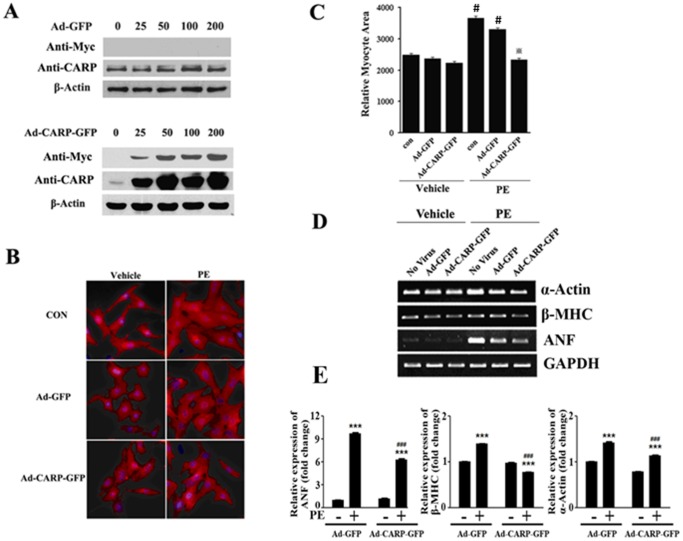
Overexpression of CARP inhibits phenylephrine-induced cardiomyocyte hypertrophy. (A) Primary neonatal rat cardiomyocytes were infected with adenoviruses expressing GFP or Myc-tagged CARP-GFP at the indicated MOIs. After 24 hours, CARP and Myc-tagged CARP levels were evaluated using Western blotting. (B) α-actin staining showed that adenoviral-mediated overexpression of CARP inhibited phenylephrine-induced cardiomyocyte hypertrophy. Cells were infected with Ad-GFP as a negative control. Nuclei were stained with DAPI. (C) Quantification of the cell surface areas shown in (B). 100–120 random cells were measured in each group. *P<0.001 relative to phenylephrine+Ad-GFP (PE+Ad-GFP), ^#^P<0.01, compared with con+vehicle; (D) The effect of CARP overexpression on the levels of mRNA of α-actin, β-MHC, and ANF in cardiomyocytes exposed to phenylephrine or not. mRNA levels were measured via semi-quantitative RT-PCR; GAPDH was employed as an internal control; (E) Quantification of mRNA levels in (D). ***P<0.001, compared with Ad-GFP, ^###^P<0.001, compared with PE+Ad-GFP.

Because the *Ankrd1* gene is upregulated in the heart in response to various hypertrophic stimuli, such as phenylephrine, endothelin, or isoproterenol, it has been hypothesized that CARP might function as a pro- or anti-hypertrophic regulator. To test this hypothesis, we first overexpressed CARP in isolated neonatal rat cardiomyocytes infected with a c-Myc-CARP-GFP-expressing recombinant adenovirus (Ad-CARP-GFP); Ad-GFP was used as a negative control. Adenoviral-mediated expression of CARP protein was confirmed by immunoblotting, using anti-CARP and anti-Myc antibodies, of cells infected with different doses of the recombinant adenovirus (25, 50, 100, and 200 MOI (Multiplicity of infection)) for 24 hours. As shown in Figure 1A, endogenous CARP protein was expressed in a dose-independent manner in Ad-GFP-infected cardiomyocytes, and no ectopically expressed CARP protein was detected in Ad-GFP-infected cardiomyocytes blotted with an anti-Myc antibody. Immunoblotting of samples from cardiomyocytes infected with Ad-CARP-GFP showed that both ectopic CARP (anti-Myc antibody) and total CARP protein (anti-CARP antibody) were expressed in an adenoviral-dose-dependent manner in these cells (Figure 1A).

To determine the effect of CARP on cardiac hypertrophy *in vitro*, we treated Ad-CARP-GFP- and Ad-GFP-infected cells, as well as non-infected cells (control), with phenylephrine (100 µM) for 24 hours to induce cardiac hypertrophy. As shown in Figure 1B and 1C, phenylephrine significantly increased the size of Ad-GFP-infected and non-infected cardiomyocytes. Importantly, we found that overexpression of CARP markedly inhibited phenylephrine-induced cardiomyocyte hypertrophy, as reflected by a decrease in myocyte area (Figure 1B and 1C) and reduced expression of the hypertrophic molecular markers α-actin, β-MHC, and ANF (Figure 1D and 1E). Together, these data indicate that overexpression of CARP in rat cardiomyocytes blocks phenylephrine-induced cardiac hypertrophy *in vitro*.

### CARP Tg Mice are Less Susceptible to Pathological Cardiac Hypertrophy Induced by Pressure Overload and Neurohumoral Stimulation Compared with Wild Type Mice

The observation that CARP attenuates cardiomyocyte hypertrophy *in vitro* prompted us to ask whether CARP might function in a similar manner *in vivo*. To investigate this possibility, we generated CARP Tg mice in which Myc-tagged CARP was selectively overexpressed in the heart under the control of the α-MHC promoter (Figure S1A). Four independent transgenic lines (B, D, E, and F) were obtained. All animals were born with the expected Mendelian ratios and were overtly normal. Transgenic mice from the F-line used in this study were characterized by PCR analysis of genomic DNA (Figure S1B) and by determination of *Ankrd1* mRNA levels (Figure S1C and S1D). To detect ectopically expressed CARP protein in transgenic mice, we analyzed total protein extracts from various tissues of 2-month-old CARP Tg mice by Western blotting using anti-Myc and anti-CARP antibodies. Figure S1E shows that ectopically expressed CARP was detected only in the hearts of CARP Tg mice. Furthermore, the level of CARP protein in the hearts of Tg mice was increased by more than 3-fold compared with that of WT mice (Figure S1F and S1G).

**Figure 2:**
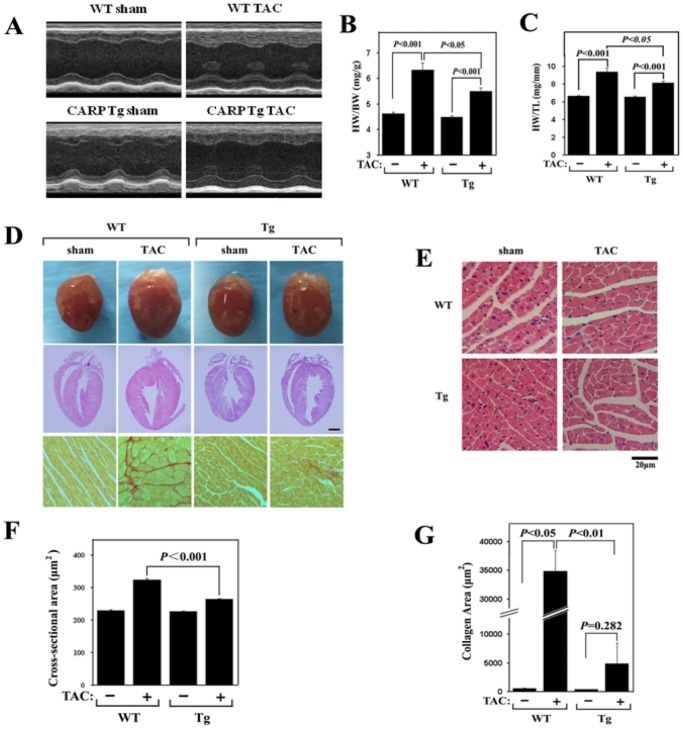
Pressure overload-induced cardiac hypertrophy is attenuated in CARP Tg mice. WT and CARP Tg mice were subjected to TAC or a sham operation. Cardiac hypertrophy and function were assessed by echocardiography. After 4 weeks, the mice were sacrificed for assessment of cardiac hypertrophy. (A) Representative examples of M-mode echocardiograms of hearts from WT and CARP Tg mice subjected to TAC or a sham operation. (B) The ratio of heart weight to body weight (HW/BW). (C) The ratio of heart weight to tibia length (HW/TL). (D) Staining of heart sections from WT and CARP Tg mice subjected to TAC or a sham operation. *Upper panel:* Gross heart morphology; *middle panel:* H&E-stained longitudinal sections; *lower panel:* PSR-stained sections. (E) H&E staining of sections from the left ventricular myocardium of WT and CARP Tg mice subjected to TAC or a sham operation. Scale bar  = 20 µm. (F) Quantification of cross-sectional areas of the cardiomyocytes shown in (E). (G) Quantification of collagen areas in the myocytes shown in (D).

Because no overt physiological abnormalities were evident in CARP Tg mice under normal growth conditions, we next investigated CARP function in pathological cardiac hypertrophy. We first assessed the hypertrophic response of WT and CARP Tg mice to a stretch stimulus, performing pressure overload using TAC in CARP Tg mice and age/gender-matched WT littermates. Echocardiographic analyses were performed to evaluate cardiac structure and function. As shown in Figure 2A and Table S1, 4 weeks after TAC, left ventricular posterior wall thickness (LVPW;d) and the LV mass of WT mice had increased by 45.5% and 47.2%, respectively, compared with those of sham-operated mice; in contrast, these two parameters were only 25.8% and 33.8% higher in TAC/CARP Tg mice than in sham-operated/CARP Tg animals. These data suggest that overexpression of CARP in the heart reduces the hypertrophic response to TAC. However, no difference in cardiac function, measured as ejection fraction percentage (EF%) and fractional shortening percentage (FS%), was evident between CARP Tg and WT mice in response to TAC.

Mice were sacrificed 4 weeks after TAC and aspects of cardiac hypertrophy were examined. The levels of CARP in hearts from wild-type TAC, transgenic sham and transgenic TAC mice were 3.05±0.20, 5.75±1.10 and 8.60±1.61 fold of that in hearts from wild-type sham mice, implying that TAC could increase CARP expression in hearts of both wild-type and transgenic mice, although showing no statistical difference (Figure S2). As shown in Figure 2B and 2C, significant increases in HW/BW and HW/TL ratios were observed in both groups compared with sham-operated controls. Interestingly, the extent of cardiac hypertrophy in CARP Tg mice was significantly lower than that in WT animals. Examination of gross heart morphology and analysis of myocyte area on histological sections after 4 weeks of TAC also revealed that the heart size was smaller and the cellular hypertrophy less in CARP Tg mice compared with WT animals (Figure 2D, 2E, and 2F). Moreover, histological analysis of the extent of fibrosis using Picric acid-Sirius red (PSR) staining showed a substantial decrease in collagen deposition in the hearts of CARP Tg mice after TAC, compared with WT animals (Figure 2D and 2G).

We also found that the expression levels of mRNAs encoding the hypertrophic markers ANF, β-MHC, and α-actin were markedly decreased in CARP Tg mice after TAC compared with the levels seen in WT animals (Figure S3A and S3C). Meanwhile the expression levels of fibrosis markers, such as procollagen type Iα2 (Col I), procollagen type III α1 (Col III), and connective tissue growth factor (CTGF), were upregulated in WT mice in response to TAC, but the effect was significantly blunted in TAC-treated CARP Tg mice (Figure S3D, S3E, and S3F). In contrast, the genes encoding myosin heavy polypeptide 6 (α-MHC) and sarco/endoplasmic reticulum Ca^2+^-ATPase 2 (SERCA2) were downregulated in WT mice in response to TAC, but the expression levels were unchanged in TAC-treated CARP Tg animals (Figure S3G and S3H).

**Figure 3:**
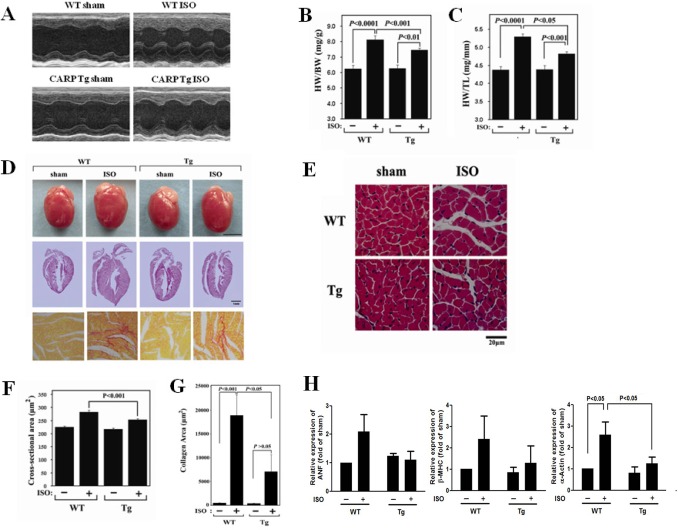
Isoproterenol-induced cardiac hypertrophy is attenuated in CARP Tg mice. WT and CARP Tg mice were continuously infused with vehicle (100 µmol/L ascorbic acid) or isoproterenol at a rate of 30 mg/kg/day using a subcutaneously implanted mini-osmotic pump. After 14 days, mice were sacrificed for assessment of cardiac hypertrophy. (A) Representative examples of M-mode echocardiograms of hearts from WT and CARP Tg mice infused with isoproterenol (ISO) or vehicle (sham). (B) The ratio of heart weight to body weight (HW/BW). (C) The ratio of heart weight to tibia length (HW/TL). (D) Staining of hearts from WT and CARP Tg mice infused with ISO or vehicle. *Upper panel:* Gross heart morphology; *middle panel:* H&E-stained longitudinal sections; *lower panel:* PSR-stained sections. (E) H&E staining of sections from the left ventricular myocardia of WT and CARP Tg mice infused with ISO or vehicle. Scale bar  = 20 µm. (F) Quantification of cross-sectional areas in the cardiomyocytes shown in (E). (G) Quantification of collagen areas in the myocytes shown in (D). (H) Changes in the expression levels of mRNAs transcribed from the ANF, β-MHC, and α-actin genes after infusion of isoproterenol in WT and CARP Tg mice. mRNA expression levels were quantitated using real-time PCR.

In addition to pressure overload stimulation, we also investigated the response of CARP Tg mice to a neurohumoral signal. We used isoproterenol to induce cardiac hypertrophy in CARP Tg mice and age/gender-matched WT littermates. After continuous infusion over 2 weeks, isoproterenol produced significant increases in LVPW;d in WT animals, as determined by echocardiography (Figure 3A, Table S2) and also increased the HW/BW and HW/TL ratios (Figure 3B and 3C), global heart size (Figure 3D), and myocyte area (Figure 3E and 3F). However, both LVPW;d and the ratios HW/BW and HW/TL were lower, and global heart size and myocyte area smaller, in CARP Tg mice than in WT animals after isoproterenol administration (Table S2 and Figure 3A–F). Furthermore, less collagen deposition was evident in the hearts of CARP Tg mice treated with isoproterenol than in the hearts of isoproterenol-treated WT animals (Figure 3D and 3G). The isoproterenol-induced increases in expression of ANF, β-MHC, and α-actin were also partially inhibited in CARP Tg mice (Figure 3H). These results indicate that overexpression of CARP markedly attenuates isoproterenol-induced cardiac hypertrophy.

Collectively, our findings provide the first experimental evidence that overexpression of CARP in the heart of the mouse attenuates cardiac hypertrophy.

### CARP Overexpression Inhibits the MAPK/ERK Signaling Pathway both *in vitro* and *in vivo*

**Figure 4:**
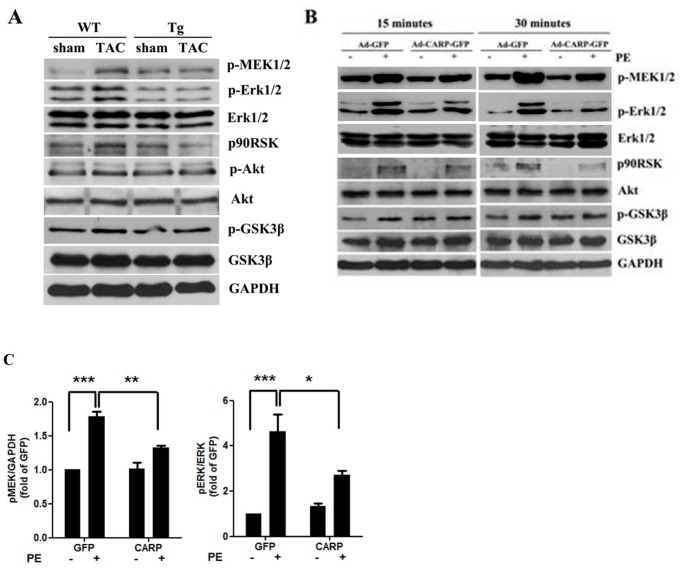
Overexpression of CARP inhibits the activation of MEK/ERK signaling by hypertrophic stimuli. (A) Western blotting of heart protein extracts to examine the levels of various phosphorylated and total kinases in WT and CARP Tg mice following TAC or a sham operation. A representative Western blot (from one of three independent experiments) is shown. (B) Western blotting of protein extracts to detect various phosphorylated and total kinase levels in parental and CARP-overexpressing cardiomyocytes subjected to phenylephrine treatment or not. A representative Western blot (from one of three independent experiments) is shown. (C) Quantification of the expression of the p-MEK1/2 and p-ERK1/2 proteins shown in (B) at 30 min after commencement of PE treatment.

To investigate the potential mechanisms by which overexpression of CARP attenuates cardiac hypertrophy, we examined several signaling pathways often involved in development of cardiac hypertrophy. The phosphoinositol 3-kinase (PI3K)/Akt and ERK signaling pathways play important roles in pressure overload-induced cardiac hypertrophy. To determine whether these signaling pathways are involved in mediation of the CARP-inhibitory action in terms of cardiac hypertrophy, we used Western blot analysis to examine the phosphorylation status of MEK1/2, ERK1/2, p90RSK, Akt, and GSK3β (all are components of the two pathways mentioned above) in hearts from TAC-treated CARP Tg mice and WT mice. As shown in Figure 4A, the most prominent changes observed affected the ERK signaling pathway. We found that the levels of phosphorylated MEK1/2, ERK1/2, and p90RSK were significantly increased in WT mice following pressure overload. Importantly, the levels of phosphorylated MEK1/2, ERK1/2, and p90RSK proteins were strikingly reduced in the hearts of CARP Tg mice (Figure 4A). However, Akt and GSK3β phosphorylation status was unaffected (Figure 4A), suggesting that the Akt signaling pathway may not participate in CARP function. To further elucidate the functional role played by the MEK1/2 MAPK signaling pathway in the cardiac hypertrophy-attenuating function of CARP, we treated CARP-overexpressing cultured neonatal rat cardiomyocytes with phenylephrine and examined activation of the ERK signaling pathway. As demonstrated in our *in vivo* experiments, the levels of phosphorylated MEK1/2, ERK1/2, and p90RSK proteins were augmented by phenylephrine treatment in a time-dependent manner in mock-transfected cells. However, phosphorylation of MEK1/2, ERK1/2, and p90RSK after phenylephrine induction was blocked in CARP-overexpressing cells (Figure 4B and 4C). Together, our findings provide *in vivo* and *in vitro* experimental evidence suggesting that the ERK signaling pathway plays a critical role in mediating the partial inhibitory effect of CARP against cardiac hypertrophy.

### Activity of the TGF-β/Smad3 Signaling Pathway in CARP Tg Mice is Decreased in Response to Pressure Overload

**Figure 5:**
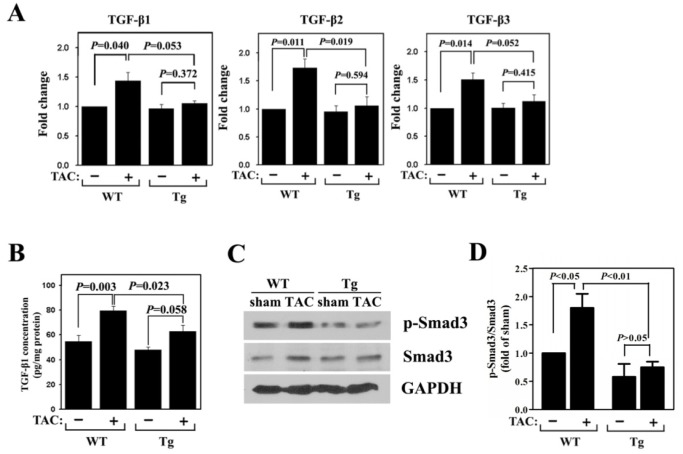
Upregulated TGF-β/Smad signaling is inhibited in CARP Tg mice following TAC. (A) Quantitative detection of TGF-β1, -β2, and -β3 mRNA expression by real-time PCR in hearts from WT and CARP Tg mice subjected to TAC or a sham operation. (B) ELISA measurements of TGF-β1 levels in heart homogenates from WT and CARP Tg mice subjected to TAC or a sham operation. (C) Western blotting to detect phosphorylated and total Smad3 protein in heart protein extracts from WT and CARP Tg mice subjected to TAC or a sham operation. (D) Quantification of the level of phosphorylated Smad 3 shown in (C).

Because TGF-β has been implicated in cardiomyocyte growth, fibrosis, and re-expression of fetal genes, we investigated whether signal transduction via this pathway was relevant to CARP function. Expressions of TGF-β1, -β2, and -β3 at the mRNA level were measured using real-time RT-PCR. All three isoforms were significantly upregulated by TAC in the hearts of WT mice (Figure 5A). However, their induction in response to TAC was blocked in the hearts of CARP Tg animals. To provide further support for a role for TGF-β in mediation of CARP function, we measured the levels of TGF-β1 content in heart homogenates. We found that the content of TGF-β1 increased significantly in WT mice after pressure overload, whereas this increase was partially attenuated in CARP Tg mice (Figure 5B).

To further determine the functional role of the TGF-β signaling pathway in the inhibitory effect of CARP on cardiac hypertrophy, we measured TGF-β pathway activation by examining the phosphorylation level of Smad3. The level of phosphorylated Smad3 protein was increased in wild-type mice after TAC surgery (Figure 5C and 5D), but this increase was abrogated in CARP Tg mice (Figure 5C and 5D).

### TGF-β1 Eliminates the Inhibitory Effect of CARP on Phenylephrine-induced Cardiomyocyte Hypertrophy

**Figure 6:**
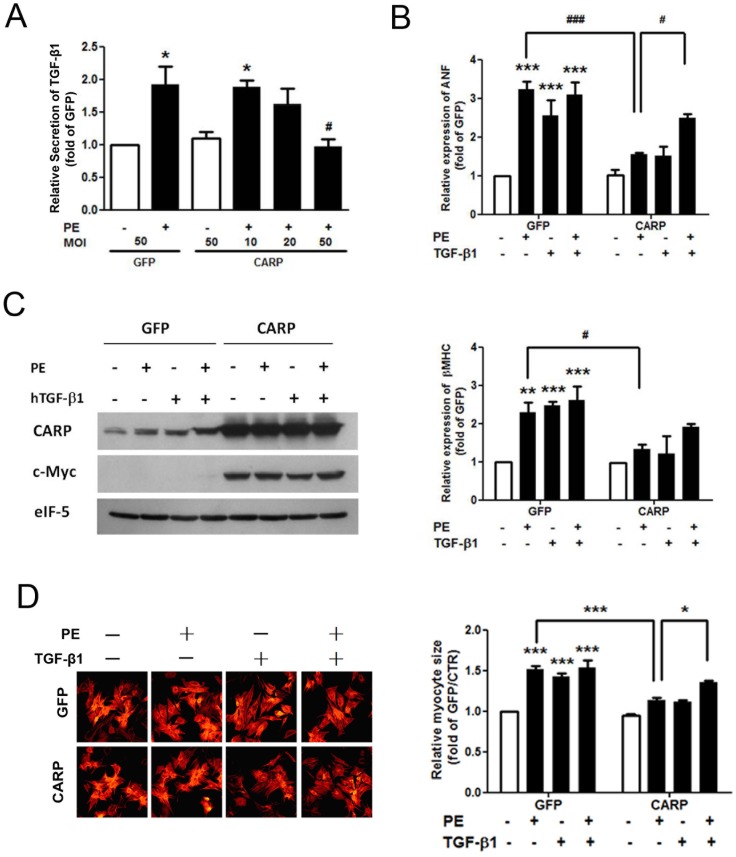
Addition of exogenous human TGF-β1 rescues the attenuation in cardiomyocyte hypertrophy evident upon CARP overexpression. (A) Overexpression of CARP inhibited phenylephrine-induced TGF-β1 secretion by cardiomyocytes. Cultured neonatal rat cardiomyocytes were infected with adenoviruses containing CARP/GFP or GFP alone and next exposed to 10 µM phenylephrine for 24 hours. The levels of TGF-β1 in culture supernatants were next determined using a double-antibody sandwich ELISA method and normalized to the concentrations in the corresponding cardiomyocytes. TGF-β1 secretion from cardiomyocytes infected with adenovirus/GFP alone was taken to be unity in each experiment. n = 4. *P<0.05, compared with the GFP control at an MOI of 50; ^#^ *P*<0.05, compared with the GFP control at an MOI of 50/PE. (B) Supplementation with exogenous TGF-β1 reversed the inhibitory effect of CARP on hypertrophic markers up-regulation in cardiomyocytes in response to phenylephrine. Cultured cardiomyocytes were infected with adenoviruses containing GFP alone or CARP/GFP/c-myc and next treated with phenylephrine, human TGF-β1, or phenylephrine plus human TGF-β1, for 24 hours. TRIzol was added and total RNA was extracted for determination of the levels of mRNA encoding ANF and β-MHC using real-time PCR. n = 3. **P<0.01, ***P<0.001, compared with GFP alone; ^#^ *P*<0.05, ^###^ *P*<0.001. (C) Western blotting to explore expression of CARP and c-myc in the cardiomyocytes of (B) above. Data from one of four independent experiments are shown. (D) The inhibitory effect of CARP overexpression on increase in cardiomyocyte size induced by phenylephrine was reversed by addition of hTFG-β1. Cultured cardiomyocytes infected with GFP or GFP/CARP adenoviruses were treated the same way as described in (B) and fixed in 4% (v/v) formaldehyde. Then F-actin was stained with Rhodamine phalloidin and myocyte size was assessed using a microscope equipped with a 200× objective and appropriate epifluorescence filters. 100 random cells were measured in each group and four independent experiments were performed. hTGF-β1, human TGF-β1; MOI, multiplicity of infection; PE, phenylephrine.

To investigate the role of TGF-β1 in mediating the effect of CARP on cardiac hypertrophy, we first examined the effect of CARP on TGF-β1 secretion from cardiomyocytes in response to a pro-hypertrophic factor, phenylephrine. As shown in Figure 6A, phenylephrine induced an approximately 1-fold increase in TGF-β1 release (compared with the baseline level) from cardiomyocytes (1.92±0.28 fold in the GFP/PE test vs 1 fold in the GFP control; *P*<0.05). However, overexpression of CARP resulted in a MOI-dependent inhibition of TGF-β1 release in response to phenylephrine; a 50% decrease was evident at an MOI of 50 compared with the level seen when an equivalent amount of control GFP-expressing virus was used for infection (0.97±0.11 fold in the CARP MOI 50/PE test vs 1.92±0.28 fold in the GFP MOI 50/PE control; *P*<0.05). Next, we investigated whether exogenous TGF-β1 could rescue the inhibitroy effects of CARP on the hypertrophic response of cardiomyocytes. Both phenylephrine and human TGF-β1 (hTGF-β1, R&D Systems) induced marked hypertrophy, as shown by expression of mRNAs encoding ANF and β-MHC (2.55±0.43- and 2.55±0.41-fold that of the GFP control, respectively, for ANF; *P*<0.01; 2.13±0.26- and 2.22±0.32-fold that of the GFP control, respectively, for β-MHC, *P*<0.01), whereas addition of hTGF-β1 plus phenylephrine did not further aggravate the hypertrophic response to phenylephrine (2.75±0.57- vs 2.55±0.43-fold for ANF and 2.14±0.35- vs 2.13±0.26-fold for β-MHC; Figure 6B). In contrast, neither phenylephrine nor hTGF-β1 induced significant hypertrophy in CARP-overexpressing cardiomyocytes (Figure 6B and 6C). However, addition of both hTGF-β1 and phenylephrine restored the hypertrophic response (2.59±0.10-fold of the GFP control for ANF and 1.91±0.08-fold of the GFP control for β-MHC) to a level similar to that in cardiomyocytes that were not transfected with CARP (2.75±0.57-fold of the GFP control level for ANF and 2.14±0.35-fold of the GFP control level for β-MHC; *P*>0.05, Figure 6B and 6C). Similarly, inhibition of phenylephrine-induced increase in cardiomyocyte size upon CARP overexpression (1.13±0.04-fold of GFP control) was also reversed to a great extent by addition of hTGF-β1(1.35±0.03-fold of GFP control; *P<0.05*, Figure 6D). Together, these results indicate that the TGF-β/Smad3 signaling pathway, in concert with the ERK MAPK pathway, may play a role in regulating CARP-mediated attenuation of cardiac hypertrophy and fibrosis in response to pressure overload *in vivo*.

### Apoptosis was not Involved in the Inhibitory Effect of CARP on Cardiac Hypertrophy

To exclude the possibility that the inhibitory effect of CARP on cardiac hypertrophy was due to apoptosis-dependent cell death, we investigated whether overexpression of CARP induced cardiomyocyte apoptosis. As shown in Figure S4A, overexpression of CARP did not cause nuclear condensation in the absence or presence of phenylephrine. Furthermore, compared with the myocytes infected with GFP alone, the levels of caspase-3 did not decrease and cleaved caspase-3 was not detected in myocytes overexpressing CARP (Figure S4B). Thus, it suggests that apoptosis does not participate in the inhibitory effect of CARP on cardiac hypertrophy.

## Discussion

Since its discovery in 1995, the *Ankrd1* gene transcript has attracted significant interest owing to its persistent upregulation in patients with cardiac hypertrophy and heart failure. However, the precise role played by the encoded CARP protein under these pathological conditions remains poorly understood. It has been proposed that inducible expression of CARP in the myocardium may indicate that the protein has a protective function, reflecting an adaptive response to various stresses [19]. To test this hypothesis, we generated cardiac-specific CARP Tg mice and investigated the functional role of CARP in cardiac hypertrophy induced by isoproterenol infusion and pressure overload. We found that overexpression of CARP markedly attenuates cardiac hypertrophy and fibrosis induced by pressure overload *in vivo*. Furthermore, we provide experimental evidence showing that this action of CARP is mediated by inhibition of the ERK1/2 and TGF-β signaling pathways. Most importantly, our findings suggest that increased CARP level may be a potential mode of prevention and treatment of cardiac hypertrophy.

It has been reported that increased expression of the *Ankrd1* gene in the left ventricular myocardium is induced by various hypertrophic stimuli, both in animal models and in heart failure patients with dilated cardiomyopathy (DCM) or ischemic cardiomyopathy (ICM) [13], [14]. Intriguingly, inducible expression of CARP also occurs in non-cardiomyocytes in response to stress. For example, the *Ankrd1* gene is upregulated in vascular endothelial cells during wound healing and promotes angiogenesis in granulation tissue [20]. Moreover, *Ankrd1* expression is strongly induced in regenerating rat skeletal muscles after damage by a single injection of bupivacaine, peaking in level 3 days after injury and falling to undetectable levels after 28 days. In addition, high-level *Ankrd1* gene expression in DMD patients is restricted to regenerating myofibers, strongly suggesting that CARP could be involved in muscle satellite cell activation during regeneration in such patients [21] **.** Functional studies have shown that overexpression of CARP in rat embryonic H9C2 cardiomyoblasts protects against hypoxia-induced apoptosis [22]. Even more striking is the observed association between CARP expression and the sensitivity of ovarian cells to cisplatin reported by Scurr et al., who showed that *Ankrd1* expression was negatively correlated with cisplatin sensitivity in a panel of human cancer cell lines and was specifically and dramatically decreased in cisplatin-sensitive lines [23]. These data indicate that, although *Ankrd1* is predominantly expressed in cardiomyocytes during normal development, its expression can be induced in non-cardiomyocytes in response to various stimuli. This stress-inducible feature of the *Ankrd1* gene, together with the evidence that CARP function is likely not restricted to cardiomyocytes, suggests that CARP is involved in a generalized response to various physiological or pathological stresses, which may be of profound cytoprotective significance. Collectively, these observations provide considerable evidence that the unique pattern of *Ankrd1* expression may reflect a general protective role of CARP in the adaptive responses of cells to stress and disease states.

In the present study, we applied 26-gauge instead of 27-gauge needle as a marker to ligate the aorta so as to exert relatively moderate pressure overload on the heart, which may simulate the course of chronic high blood pressure better. Similar to the previous work reported in our lab [16], the method did make a successful TAC model because: 1) Echocardiographic analysis showed that the blood flow velocity at the ligation site in the TAC mice markedly increased compared with that in sham-operated mice (2980.4±406.3 mm/s for WT-TAC and 3279.7±498.6 mm/s for Tg-TAC vs. 756.4±106.5 mm/s for WT-sham and 877.9±119.3 mm/s for Tg-sham, respectively, *P*<0.01, Figure S5); 2) Left ventricular hypertrophy was usually apparent at 3 weeks after TAC surgery as determined by echocardiogram (0.81±0.02 mm for WT-TAC vs. 0.65±0.02 mm for WT-sham as indicated by LVPW;d, data not shown), and became more severe at 4 weeks after TAC (Table S1); 3) Histological staining showed increased myocyte size and interstitial fibrosis; 4) Hypertrophic markers altered significantly, and etc. Since the aorta was moderately constricted, although cardiac hypertrophy happened soon after TAC surgery, the heart might gradually adapt to the stress and keep at the compensating state for quite a long period. This kind of model might better mimic the course of cardiac hypertrophy resulting from chronic high blood pressure, but could not progress into heart failure within a short time like some TAC surgeries which constriction is carried out against a 27-gauge needle or to a greater extend [24], [25]. Although we could not observe if CARP protected against heart failure during a relatively short period, there were still some clues showing that CARP may play a protective role since overexpression of CARP decreased fibrosis deposition in heart and did not result in cardiomyocyte apoptosis. Besides, Cinquetti et al. found that CARP gene expression and mutation were evident in lymphoblastoid cell lines derived from both a translocation-bearing proband and an independent sporadic total anomalous pulmonary venous return (TAPVR) patient [26]. The study shed light on the functional role played by CARP in cardiomyopathies and suggested that *Ankrd1* gene therapy or modulation of CARP activity could be viable therapeutic strategies against different types of cardiomyopathies.

Several signaling pathways have been implicated in the regulation of cardiac hypertrophy; these include the Raf/MEK/MAPK, PI3K/Akt, and JAK/STAT pathways [27]. It has been reported that three major MAPK pathways, ERK, SAPK/JNK (stress-activated/c-Jun N-terminal kinase), and p38 MAPK, are activated in the cardiac tissue of mice following TAC surgery [28]. ERK plays an essential role in mediating the cardiomyocyte hypertrophy induced by the hypertrophic agonists endothelin-1 and phenylephrine [29], [30]. In the present study, we found that phosphorylation levels of MEK1/2, ERK1/2, and their downstream target, ribosomal S6 kinase (p90RSK), were remarkably increased after TAC in WT mice, but were unchanged in CARP Tg mice (Figure 6). In contrast, we observed no significant differences in p38, Stat3, or Akt phosphorylation levels between WT and CARP Tg mice subjected to TAC (data not shown). Thus, our data suggest that CARP alleviates cardiac hypertrophy, at least in part, via inhibition of the MEK1/2/ERK1/2 pathway. However, activation of the MEK1/2/ERK1/2 pathway appears to contribute only to cardiomyocyte hypertrophy and not to interstitial fibrosis.

Because TGF-β controls the expression of both ECM network components, such as the fibrillar collagens and fibronectin, and also protease inhibitors, including PAI-1 and TIMPs. TGF-β is an important regulator of extracellular matrix (ECM) deposition. These actions render TGF-β central to the development of tissue fibrosis. TGF-β1, the most important isoform in the cardiovascular system, has been reported to play a central role in the development of heart hypertrophy and heart failure. Transgenic mice overexpressing TGF-β1 display prominent cardiac hypertrophy caused by increases in both cardiomyocyte growth and intercellular fibrosis. We have shown that pressure overload-induced hypertrophy was partially inhibited in CARP Tg mice and, most importantly, that TGF-β1, TGF-β2, and TGF-β3 levels were decreased compared with those of WT animals. In addition, fibrotic marker expression levels were significantly decreased in CARP Tg mice after pressure overload, indicating that expression of the genes encoding collagen synthesis was blocked in the hearts of CARP transgenic mice in response to TAC. This observation is consistent with the results of PSR staining of ventricular heart tissue. Because we also found that TGF-β protein levels were downregulated in CARP Tg mice, it is reasonable to propose that the reduced expression and secretion of TGF-β in such animals, in response to TAC, may contribute to CARP functions in attenuating cardiomyocyte hypertrophy. Our experimental evidence supports this notion. Indeed, TGF-β1 secretion was significantly decreased in CARP-overexpressing cardiomyocytes, in a manner that appeared to be dependent on the MOI of the CARP-expressing viral vector. However, infection with adenovirus carrying GFP alone did not affect the TGF-β1 release level, suggesting that the decrease in TGF-β1 secretion is CARP-dependent. In addition, CARP overexpression inhibited cardiomyocyte hypertrophy in response to phenylephrine. Notably, addition of exogenous TGF-β1 (hTGF-β1) reversed this inhibitory effect of CARP, indicating that the TGF-β signaling pathway participates in the inhibitory action of CARP in terms of cardiac hypertrophy. It is worth mentioning that hTGF-β1 (16 ng/mL) also induced cardiomyocyte hypertrophy and that this effect was also inhibited when CARP was overexpressed. This may be due to that CARP functions as a negative regulator which can directly inhibit quite a few pro-hypertrophic factors, including the downstream molecules of TGF-β1, so addition of hTGF-β1 alone could not reverse the inhibitory effect of CARP on cardiomyocyte hypertrophy. However, when hTGF-β1 and phenylephrine act together, some unknown synergistic mechanism may be triggered and thereby partly reversing the negative effect of CARP on hypertrophy.

Further support for the proposed deficiency in TGF-β function is provided by our finding that the levels of phosphorylated Smad3, a downstream target of TGF-β signaling, were significantly decreased in the hearts of CARP Tg mice. Smads play central roles in regulating ECM gene expression in response to TGF-β, and Smad3 mediates acute and chronic changes in gene expression that leads to inflammation and fibrosis. Smad3 also plays a role in mediating the effects of angiotensin II; chronic exposure to this protein promotes a profibrotic environment via multiple mechanisms, including increased expression of TGF-β1 and stimulation of Smad3-dependent gene expression.

Since apoptosis causes cell death and cell loss, which directly result in thinning of ventricular wall and heart failure without compensating hypertrophy, we asked whether CARP attenuated cardiac hypertrophy via activating apoptosis. However, our results showed that overexpression of CARP did not promote nuclear condensation and activation of caspase-3 pathway, which are regarded as the distinctive features of apoptosis at biochemical level [31]. The findings suggest that apoptosis is not involved in the inhibitory effect of CARP on cardiac hypertrophy.

It is well-known that analysis of genetically engineered mice has great advantages for our understanding of the in vivo function of a given gene and protein. In the present study we applied cardiac-specific transgenic mice as a model to investigate the function of CARP in cardiac hypertrophy. Cardiac-specific transgene can exclude the impacts of CARP overexpressed in other tissues or organs, and as a negative regulator, gain-of-function study (transgenic model) seems more suitable for investigation of CARP function. However, since the level of CARP in transgenic mice is much higher than normal level, some non-specific effects would inevitably occur.

In summary, our data provide the first evidence that overexpression of CARP decreases the activity of both the ERK1/2 and the TGF-β/Smads signaling pathways and subsequently attenuates cardiac hypertrophy and fibrosis in the hearts of CARP transgenic mice (Figure S6). Our findings thus provide compelling support for a role for CARP in inhibiting cardiac hypertrophy and fibrosis. More importantly, our results highlight the potential significance of CARP as an anti-hypertrophic factor with therapeutic potential against cardiac hypertrophy in humans.

## Supporting Information

Figure S1 **Establishment and identification of cardiac-specific CARP Tg mice.** (A) Schematic diagram of the α-MHC-CARP plasmid. (B) Distinguishing CARP transgenic (Tg) mice from wild-type (WT) mice by PCR genotyping. (C) Expression of CARP in CARP Tg and WT mice as detected by semi-quantitative RT-PCR. GAPDH was used as an internal control. (D) Quantification of the CARP expression shown in (C). (E) Expression of CARP and CARP-Myc fusion proteins in the hearts of WT and CARP Tg mice as detected by Western blotting. β-tubulin was used as an internal control. (F) Quantification of the CARP expression shown in (E). (G) Tissue-specific expression of transgenic CARP in the heart, relative to other tissues (as indicated).
(TIF)

Figure S2 **Relative levels of CARP in hearts from wild-type and CARP transgenic mice subjected to sham-operation or TAC.** Heart tissue lysates were separated with electrophoresis and the relative levels of CARP and c-myc were detected by Western blotting. Quantification of CARP expression was also shown here. The data were representative of 2 separate experiments (4 samples for each group, i.e. n = 4).
(TIF)

Figure S3 **Molecular markers of cardiac hypertrophy are inversely regulated in CARP Tg mice in response to pressure-overload.** Expression of mRNAs encoding molecular markers in the hearts of WT and CARP Tg mice subjected to TAC or a sham operation were detected using real-time PCR. Col I, procollagen type Iα2; Col III, procollagen type III α1; Myh6, α-MHC.
(JPG)

Figure S4 **Overexpression of CARP did not induce apoptosis of cardiomyocytes.** Cardiomyocytes infected with indicated adenovirus constructs were incubated for 48 hours in serum-free medium and treated with or without phenylephrine for 24 hours then: (A) The cells were fixed and stained for nuclear chromatin with Hochest 33342. Fluorescent confocal micrographs were obtained using 2 different filters to visualize GFP or GFP-CARP expression (top), nucleus (medium) and overlapped image (bottom) without changing the viewing field. Note that neither nuclear chromatin nor karyorrhexis occurred in CARP-overexpressed myocytes treated with or without phenylephrine. Scale bar = 50 µm, from 100 infected cells for each of the treatment condition. (B) Cardiomyocytes were collected and the levels of caspase-3 were assessed by Western blotting. Neither decrease in caspase-3 expression nor cleaved caspase-3 was detected in CARP-overexpressed cells. Data are representative of 3 separate experiments.
(TIF)

Figure S5 **Representative examples of Doppler echocardiography detecting the aortic blood flow at the ligation site of TAC surgery.** The wild type and CARP Tg mice were subjected to TAC or sham-operation. Four weeks later, the mice were assessed by echocardiography under anesthesia. The Doppler images showed velocity of aortic blood flow at ligation site were much higher in the mice subjected to TAC than that in sham-operated mice.
(TIF)

Figure S6 **A Schematic Model for CARP Function in Protecting Cardiac Hypertrophy and Fibrosis.**
(TIF)

Table S1 **Echocardiographic analysis of LV remodeling in response to TAC in CARP Tg mice and WT littermates.** All values are means ± SEMs. EF, ejection fraction; FS, fractional shortening; HR, heart rate; LVID;d, end-diastolic left ventricular internal dimension; LVID;s, end-systolic left ventricular internal dimension; LV mass, left ventricular mass, which equals to 1.053*[(LVID;d+LVPW;d+LVAW;d)^3^-LVID;d^3^]*0.8; LVPW;d, end-diastolic left ventricular posterior wall; LVPW;s, end-systolic left ventricular posterior wall; LVAW;d, end-diastolic left ventricular anterior wall; LVAW;s, end-systolic left ventricular anterior wall;. ^**^ *P*<0.01, ^***^ *P*<0.001, compared to sham-operated mice; ^#^ *P*<0.05, ^###^ *P*<0.001, compared to WT mice subjected to TAC.
(DOC)

Table S2 **Echocardiographic analysis of LV remodeling in response to isoproterenol infusion in CARP Tg mice and WT littermates.** All values shown are means ± SEMs. EF, ejection fraction; FS, fractional shortening; HR, heart rate; ISO, isoproterenol; LVID;d, end-diastolic left ventricular internal dimension; LVID;s, end-systolic left ventricular internal dimension; LV mass, left ventricular mass, which equals to 1.053*[(LVID;d+LVPW;d+LVAW;d)^3^-LVID;d^3^]*0.8; LVPW;d, end-diastolic left ventricular posterior wall; LVPW;s, end-systolic left ventricular posterior wall; LVAW;d, end-diastolic left ventricular anterior wall; LVAW;s, end-systolic left ventricular anterior wall. ^**^ *P*<0.01, ^***^ *P*<0.001, compared to vehicle-infused mice; ^##^ *P*<0.01, ^###^ *P*<0.001, compared to WT mice treated with ISO.
(DOC)

Supporting Information S1. (DOC)
