# Supplementary figures and images for: Cardiac Ankyrin Repeat Protein Attenuates Cardiac Hypertrophy by Inhibition of ERK1/2 and TGF-β Signaling Pathways
Source: PLoS One. 2012 Dec 5;7(12):e50436. doi: 10.1371/journal.pone.0050436 (PMC3515619; doi:10.1371/journal.pone.0050436)

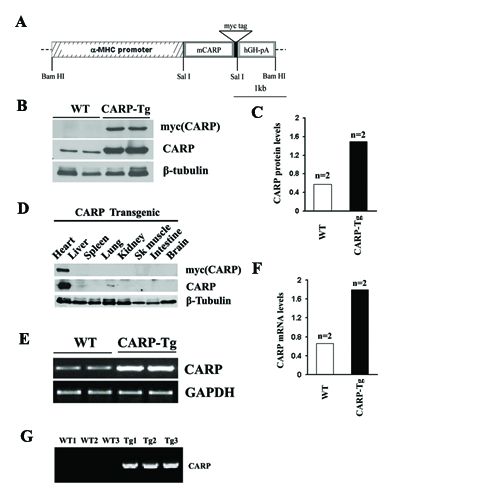

Supplement: Figure S1 — Establishment and identification of cardiac-specific CARP Tg mice. (A) Schematic diagram of the α-MHC-CARP plasmid. (B) Distinguishing CARP transgenic (Tg) mice from wild-type (WT) mice by PCR genotyping. (C) Expression of CARP in CARP Tg and WT mice as detected by semi-quantitative RT-PCR. GAPDH was used as an internal control. (D) Quantification of the CARP expression shown in (C). (E) Expression of CARP and CARP-Myc fusion proteins in the hearts of WT and CARP Tg mice as detected by Western blotting. β-tubulin was used as an internal control. (F) Quantification of the CARP expression shown in (E). (G) Tissue-specific expression of transgenic CARP in the heart, relative to other tissues (as indicated). (TIF) [file pone.0050436.s001.tif]

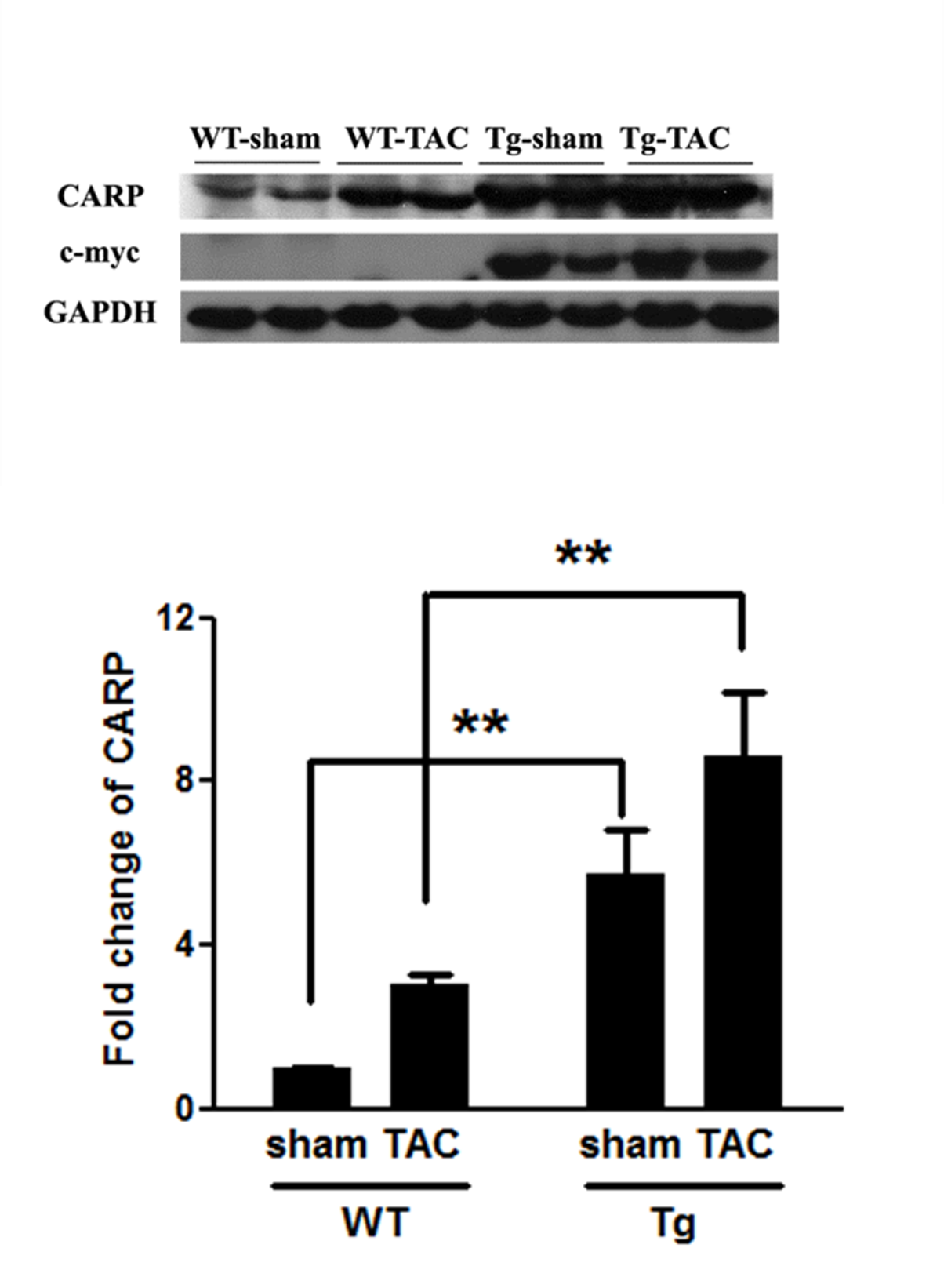

Supplement: Figure S2 — Relative levels of CARP in hearts from wild-type and CARP transgenic mice subjected to sham-operation or TAC. Heart tissue lysates were separated with electrophoresis and the relative levels of CARP and c-myc were detected by Western blotting. Quantification of CARP expression was also shown here. The data were representative of 2 separate experiments (4 samples for each group, i.e. n = 4). (TIF) [file pone.0050436.s002.tif]

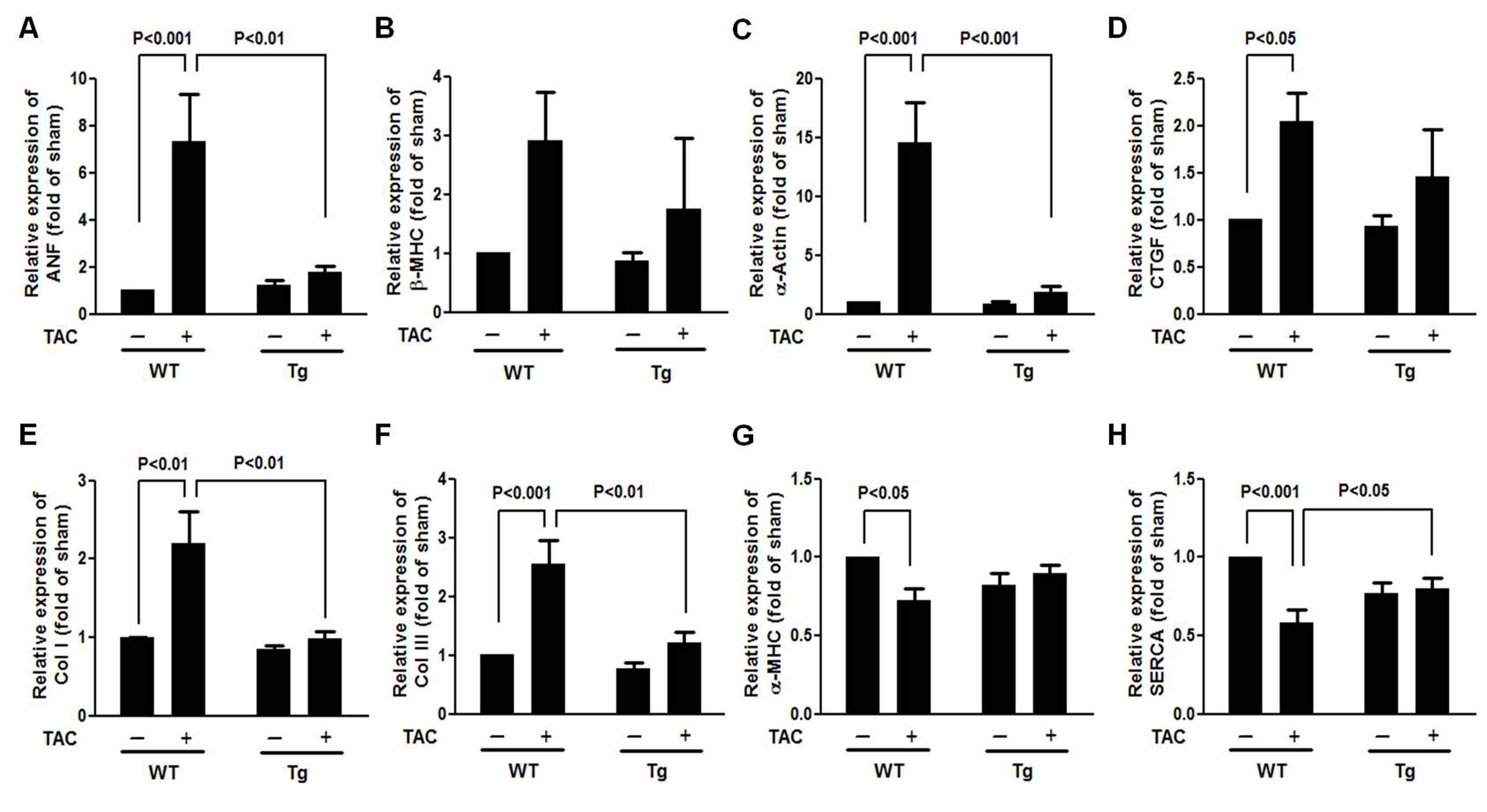

Supplement: Figure S3 — Molecular markers of cardiac hypertrophy are inversely regulated in CARP Tg mice in response to pressure-overload. Expression of mRNAs encoding molecular markers in the hearts of WT and CARP Tg mice subjected to TAC or a sham operation were detected using real-time PCR. Col I, procollagen type Iα2; Col III, procollagen type III α1; Myh6, α-MHC. (JPG) [file pone.0050436.s003.jpg]

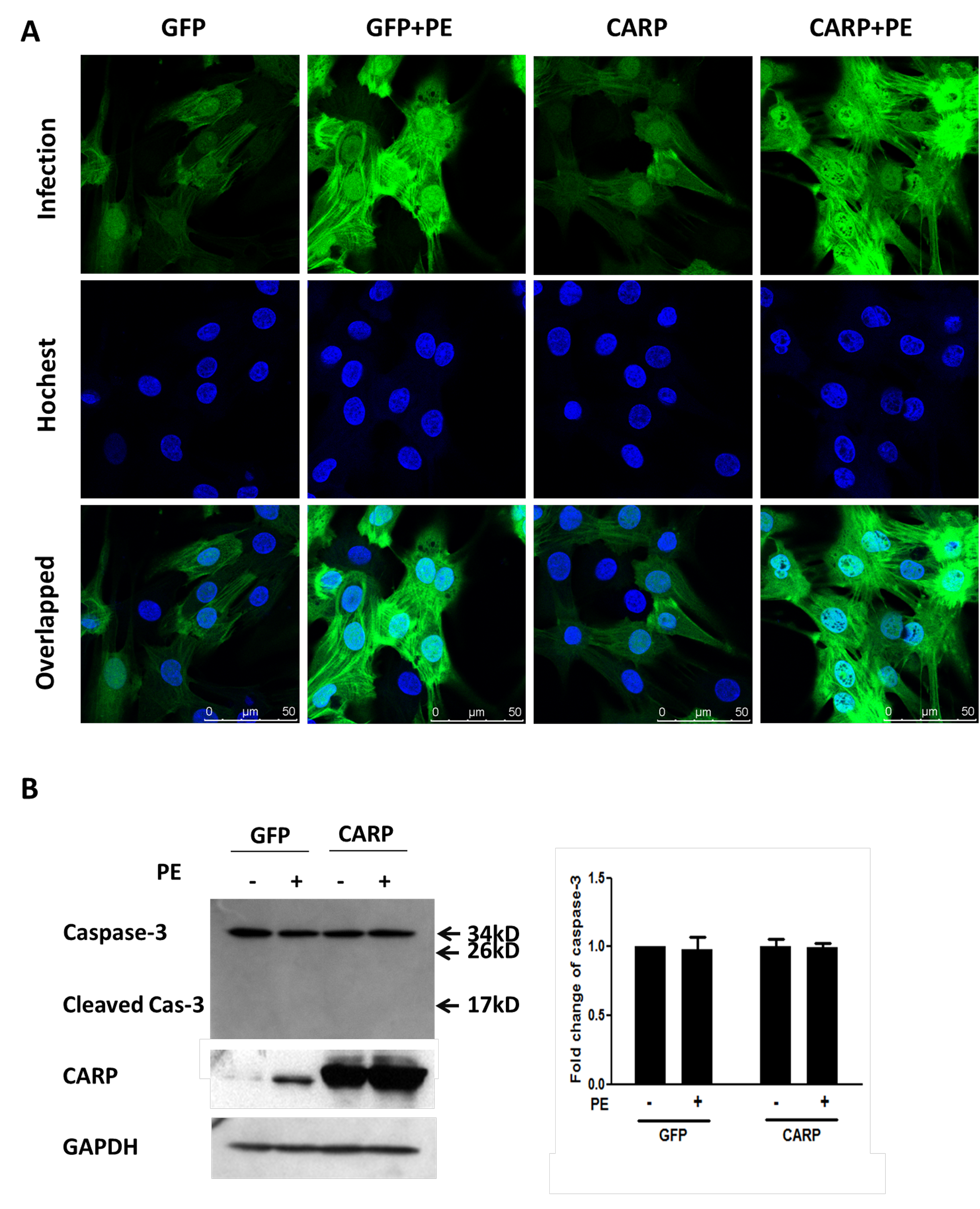

Supplement: Figure S4 — Overexpression of CARP did not induce apoptosis of cardiomyocytes. Cardiomyocytes infected with indicated adenovirus constructs were incubated for 48 hours in serum-free medium and treated with or without phenylephrine for 24 hours then: (A) The cells were fixed and stained for nuclear chromatin with Hochest 33342. Fluorescent confocal micrographs were obtained using 2 different filters to visualize GFP or GFP-CARP expression (top), nucleus (medium) and overlapped image (bottom) without changing the viewing field. Note that neither nuclear chromatin nor karyorrhexis occurred in CARP-overexpressed myocytes treated with or without phenylephrine. Scale bar = 50 µm, from 100 infected cells for each of the treatment condition. (B) Cardiomyocytes were collected and the levels of caspase-3 were assessed by Western blotting. Neither decrease in caspase-3 expression nor cleaved caspase-3 was detected in CARP-overexpressed cells. Data are representative of 3 separate experiments. (TIF) [file pone.0050436.s004.tif]

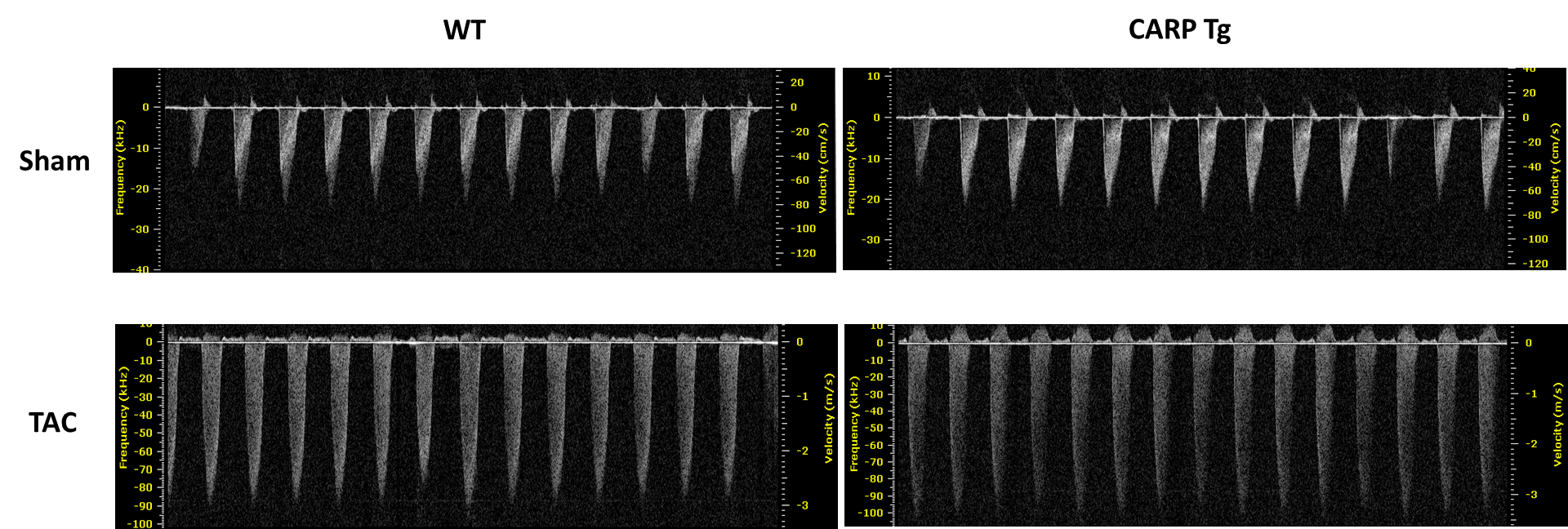

Supplement: Figure S5 — Representative examples of Doppler echocardiography detecting the aortic blood flow at the ligation site of TAC surgery. The wild type and CARP Tg mice were subjected to TAC or sham-operation. Four weeks later, the mice were assessed by echocardiography under anesthesia. The Doppler images showed velocity of aortic blood flow at ligation site were much higher in the mice subjected to TAC than that in sham-operated mice. (TIF) [file pone.0050436.s005.tif]
