# Supplementary material for: Cardiac Ankyrin Repeat Protein Attenuates Cardiac Hypertrophy by Inhibition of ERK1/2 and TGF-β Signaling Pathways
Source: PLoS One. 2012 Dec 5;7(12):e50436. doi: 10.1371/journal.pone.0050436 (PMC3515619; doi:10.1371/journal.pone.0050436)
Supplement: Table S1 — Echocardiographic analysis of LV remodeling in response to TAC in CARP Tg mice and WT littermates. All values are means ± SEMs. EF, ejection fraction; FS, fractional shortening; HR, heart rate; LVID;d, end-diastolic left ventricular internal dimension; LVID;s, end-systolic left ventricular internal dimension; LV mass, left ventricular mass, which equals to 1.053*[(LVID;d+LVPW;d+LVAW;d)3-LVID;d3]*0.8; LVPW;d, end-diastolic left ventricular posterior wall; LVPW;s, end-systolic left ventricular posterior wall; LVAW;d, end-diastolic left ventricular anterior wall; LVAW;s, end-systolic left ventricular anterior wall;. ** P<0.01, *** P<0.001, compared to sham-operated mice; # P<0.05, ### P<0.001, compared to WT mice subjected to TAC. (DOC) [file pone.0050436.s007.doc]

**Table S**1. Echocardiographic analysis of LV remodeling in response to TAC in CARP Tg mice and WT littermates.

|  | WT | | CARP Tg | |
| --- | --- | --- | --- | --- |
|  | Sham | TAC | Sham | TAC |
|  | n = 10 | n = 11 | n = 9 | n = 10 |
| LVPW;d (mm) | 0.66±0.01 | 0.96±0.03^***^ | 0.66±0.01 | 0.83±0.03^***###^ |
| LVPW;s (mm) | 0.98±0.02 | 1.28±0.03^***^ | 0.96±0.03 | 1.18±0.04^***#^ |
| LVAW;d (mm) | 0.67±0.01 | 0.92±0.02^***^ | 0.67±0.01 | 0.82±0.02^***###^ |
| LVAW;s (mm) | 1.01±0.02 | 1.27±0.04^***^ | 0.97±0.03 | 1.19±0.03^***^ |
| LVID;d (mm) | 4.10±0.26 | 3.90±0.23 | 4.05±0.14 | 4.03±0.35 |
| LVID;s (mm) | 3.10±0.36 | 3.01±0.34 | 3.16±0.27 | 2.94±0.51 |
| EF (%) | 48.94±2.58 | 46.44±2.84 | 44.63±3.36 | 49.88±3.03 |
| FS (%) | 24.59±1.52 | 23.03±1.60 | 22.11±1.96 | 27.35±2.71 |
| LV mass (mg) | 77.31±3.29 | 113.84±6.20^***^ | 74.93±1.92 | 99.53±4.47^**^ |
| HR (bpm) | 429±19 | 418±11 | 407±12 | 430±15 |

All values are means ± SEMs. EF, ejection fraction; FS, fractional shortening; HR, heart rate; LVID;d, end-diastolic left ventricular internal dimension; LVID;s, end-systolic left ventricular internal dimension; LV mass, left ventricular mass, which equals to 1.053*[(LVID;d+LVPW;d+LVAW;d)^3^-LVID;d^3^]*0.8; LVPW;d, end-diastolic left ventricular posterior wall; LVPW;s, end-systolic left ventricular posterior wall; LVAW;d, end-diastolic left ventricular anterior wall; LVAW;s, end-systolic left ventricular anterior wall;. ^**^*P* < 0.01, ^***^*P* < 0.001, compared to sham-operated mice; ^#^*P* < 0.05, ^###^*P* < 0.001, compared to WT mice subjected to TAC.
