# Supplementary material for: Cardiac Ankyrin Repeat Protein Attenuates Cardiac Hypertrophy by Inhibition of ERK1/2 and TGF-β Signaling Pathways
Source: PLoS One. 2012 Dec 5;7(12):e50436. doi: 10.1371/journal.pone.0050436 (PMC3515619; doi:10.1371/journal.pone.0050436)
Supplement: Table S2 — Echocardiographic analysis of LV remodeling in response to isoproterenol infusion in CARP Tg mice and WT littermates. All values shown are means ± SEMs. EF, ejection fraction; FS, fractional shortening; HR, heart rate; ISO, isoproterenol; LVID;d, end-diastolic left ventricular internal dimension; LVID;s, end-systolic left ventricular internal dimension; LV mass, left ventricular mass, which equals to 1.053*[(LVID;d+LVPW;d+LVAW;d)3-LVID;d3]*0.8; LVPW;d, end-diastolic left ventricular posterior wall; LVPW;s, end-systolic left ventricular posterior wall; LVAW;d, end-diastolic left ventricular anterior wall; LVAW;s, end-systolic left ventricular anterior wall. ** P<0.01, *** P<0.001, compared to vehicle-infused mice; ## P<0.01, ### P<0.001, compared to WT mice treated with ISO. (DOC) [file pone.0050436.s008.doc]

**Table S2. Echocardiographic analysis of LV remodeling in response to isoproterenol infusion in CARP Tg mice and WT littermates.**

|  | WT | | CARP Tg | |
| --- | --- | --- | --- | --- |
|  | Vehicle | ISO | Vehicle | ISO |
|  | (n = 9) | (n = 8) | (n = 7) | (n = 9) |
| LVPW;d (mm) | 0.68±0.01 | 0.90±0.02^***^ | 0.65±0.01 | 0.80±0.01^***###^ |
| LVPW;s (mm) | 1.08±0.03 | 1.39±0.04^***^ | 1.01±0.02 | 1.24±0.03^***##^ |
| LVAW;d (mm) | 0.68±0.01 | 0.91±0.03^***^ | 0.67±0.01 | 0.80±0.02^***###^ |
| LVAW;s (mm) | 1.03±0.03 | 1.35±0.04^***^ | 1.05±0.03 | 1.21±0.03^***##^ |
| LVID;d (mm) | 3.72±0.12 | 3.90±0.29 | 3.97±0.23 | 3.91±0.33 |
| LVID;s (mm) | 2.55±0.19 | 2.70±0.31 | 2.88±0.27 | 2.67±0.37 |
| FS (%) | 30.74±2.57 | 34.16±1.66 | 27.38±1.37 | 31.15±0.98 |
| LV mass (mg) | 68.73±4.65 | 105.45±6.98^***^ | 72.61±2.75 | 91.37±3.46^*^ |
| HR (bpm) | 434±16 | 521±19^**^ | 422±14 | 533±21^***^ |

All values shown are means ± SEMs. EF, ejection fraction; FS, fractional shortening; HR, heart rate; ISO, isoproterenol; LVID;d, end-diastolic left ventricular internal dimension; LVID;s, end-systolic left ventricular internal dimension; LV mass, left ventricular mass, which equals to 1.053*[(LVID;d+LVPW;d+LVAW;d)^3^-LVID;d^3^]*0.8; LVPW;d, end-diastolic left ventricular posterior wall; LVPW;s, end-systolic left ventricular posterior wall; LVAW;d, end-diastolic left ventricular anterior wall; LVAW;s, end-systolic left ventricular anterior wall. ^**^*P*<0.01, ^***^*P*<0.001, compared to vehicle-infused mice; ^##^*P* < 0.01, ^###^*P* < 0.001, compared to WT mice treated with ISO.
