# Supplementary material for: Cardiac Ankyrin Repeat Protein Attenuates Cardiac Hypertrophy by Inhibition of ERK1/2 and TGF-β Signaling Pathways
Source: PLoS One. 2012 Dec 5;7(12):e50436. doi: 10.1371/journal.pone.0050436 (PMC3515619; doi:10.1371/journal.pone.0050436)
Supplement: Supporting Information S1. — (DOC) [file pone.0050436.s009.doc]

**Supplementary Materials for:**

**Cardiac ankyrin repeat protein attenuates cardiac hypertrophy by**

**inhibition of ERK1/2 and TGF-**β **signaling pathways**

**Yao Song, PhD; Jialin Xu, PhD; Yanfeng Li, PhD; Chunshi Jia, MD, PhD;**

**Xiaowei Ma, PhD; Lei Zhang; Yong Zhang, PhD; Xiang Gao, PhD; Youyi**

**Zhang, MD, PhD; Dahai Zhu, PhD**

**Methods**

**Generation of transgenic mice**

A CARP expression construct was prepared by cloning a 960-bp cDNA containing the coding region of mouse CARP downstream of the alpha-mouse cardiac myosin heavy chain (α-MHC) promoter. Transgenic mice were produced by microinjection of the α-MHC-CARP construct into fertilized mouse embryos (129 strain background). Four independent transgenic lines (B, D, E, and F) were established. The F-line transgenic mouse was backcrossed with C57/BL6J animals for more than five generations to create a >98.5% C57/BL6J background. Transgenic mice were identified by PCR analysis of genomic DNA using the forward primer 5’-CAT GCT TAG AAG GAC ACT TGG C-3’ and the reverse primer 5’-CAC AGA TCC TCT TCT GAG ATG-3’. Transgenic expression of CARP in the heart was confirmed by Western blotting (see below).

**Histological analysis**

Hearts were collected, fixed in 4% (v/v) paraformaldehyde (PBS-buffered), dehydrated, and embedded in paraffin. Heart sections (5 μm in thickness) were stained with hematoxylin and eosin (H&E) for routine histological examination; Picrosirius red (PSR) was used to stain collagen. Cell and collagen areas were quantified in hearts from four animals of each experimental group using a Leica Q550IW imaging analytical system. Cell area was quantified in at least 80 myocytes (×400 magnification) and collagen was quantified in five visual fields (×40 magnification).

**Immunocytochemistry and cell size measurements**

Cardiomyocytes grown on laminin-coated cover slips were infected with recombinant adenovirus for 24 hours and then treated with 100 μM phenylephrine for 24 hours. After infection, cells were fixed in 4% (v/v) formaldehyde for 10 minutes, permeabilized with 0.5% (v/v) Triton X-100 in PBS for 5 minutes, and blocked via incubation in a 5% (w/v) BSA solution for 1 hour at room temperature. The cells were first incubated with an anti-α-actin antibody (Sigma) and then with tetramethyl rhodamine isothiocyanate-conjugated goat anti-mouse IgG. Cells were next mounted using Vectashield mounting medium. For assessing the effect of TGF-β1 on cardiomyocytes size in the absence or presence of CARP overexpression, the cells were dyed with Rhodamine phalloidin to show F-actin for 60 min at 37 °C. Immunofluorescence or Rhodamine was analyzed using a microscope equipped with a 200× objective and appropriate epifluorescence filters (Olympus, Tokyo, Japan). Areas were measured employing NIH ImageJ software.

**Quantification of TGF-β1 protein production**

The concentrations of TGF-β1 in heart homogenates and supernatants of cultured neonatal rat cardiomyocytes were measured using a specific ELISA kit (Shanghai ExCell Biology, Inc., Shanghai, China). Briefly, the homogenates or culture supernatants were first activated by incubation with 1 M HCl for 1 hour followed by neutralization with 1 M NaOH. Activated samples were next transferred to microwell plates coated with anti-TGF-β1 antibody. After incubation for 1.5 hours at 37 °C and thorough washing, biotin-conjugated antibody directed against TGF-β1 was added for an 1 hour’s incubation, followed by an incubation with streptavidin HRP for 30 min at 37 °C. Excess antibody or streptavidin-HRP was removed by washing and an HRP substrate was added. OD_450_ values were measured using a Microplate reader (Model 680, Bio-Rad). The concentrations of TGF-β1 were calculated using a standard curve and normalized to the total protein level.

**Western blotting**

Cardiac tissue and cultured cardiomyocytes were lysed in 1× RIPA lysis buffer (50 mM Tris, 150 mM NaCl, 1% [v/v] Triton X-100, and 0.5% [w/v] deoxycholate) containing 25 μg/mL leupeptin, 10 μg/mL aprotinin, 1 mM sodium orthovanadate, and 2 mM EDTA. The protein concentrations of all samples were determined using a Bio-Rad Protein Assay Reagent kit (BioRad). Cell lysate proteins (50 μg) were resolved on 12% (w/v) SDS-PAGE and next transferred to Immobilon-P membranes (Millipore, Billerica, MA). After blocking with 10% (w/v) nonfat milk at room temperature for 1 hour, membranes were incubated with primary antibodies at 4°C overnight, followed by incubation with horseradish peroxidase-conjugated secondary antibodies. Blots were developed using an enhanced chemiluminescence detection kit (ECL; Pierce, Rockford, IL).

**Primer sequences used for real-time PCR**

Forward and reverse primers for GAPDH were 5’-TGGAGAAACCTGCCAAGTATGA-3’ and 5’-CTGTTGAAGTCGCAGGAGACA-3’.

Forward and reverse primers for rat ANF were 5’- AGTGCGGTGTCCAACACAGA-3’ and 5’- TCTCCTCCAGGTGGTCTAGCA-3’.

Forward and reverse primers for rat α-Actin were 5’- CGCGACCTTACTGACTACCTG-3’ and 5’- TCTCACGTTCAGCTGTGGTC-3’.

Forward and reverse primers for rat CARP were 5’- GAGCTGGTAACGGGCAAAAA-3’ and 5’- GAACTCGCCAGGAAGGAACTC-3’.

Forward and reverse primers for mouse ANF were 5’- GCTTCCAGGCCATATTGGAG-3’ and 5’- GGGGGCATGACCTCATCTT-3’.

Forward and reverse primers for mouse α-MHC were 5’- GCCCAGTACCTCCGAAAGTC-3’ and 5’- GCCTTAACATACTCCTCCTTGTC-3’.

Forward and reverse primers for mouse β-MHC were 5’- ACTGTCAACACTAAGAGGGTCA-3’ and 5’- TTGGATGATTTGATCTTCCAGGG-3’.

Forward and reverse primers for mouse α-Actin were 5’- GCGTGGCTATTCCTTCGTGA-3’ and 5’- TGGCCATCTCATTCTCGAAGT-3’.

Forward and reverse primers for mouse SERCA were 5’- GAGAACGCTCACACAAAGACC-3’ and 5’- CAATTCGTTGGAGCCCCAT-3’.

Forward and reverse primers for mouse CTGF were 5’- GGGCCTCTTCTGCGATTTC-3’ and 5’- ATCCAGGCAAGTGCATTGGTA-3’.

Forward and reverse primers for mouse Col I were 5’- GTAACTTCGTGCCTAGCAACA-3’ and 5’- CCTTTGTCAGAATACTGAGCAGC-3’.

Forward and reverse primers for mouse Col III were 5’- CCTGGCTCAAATGGCTCAC-3’ and 5’- CAGGACTGCCGTTATTCCCG-3’.

Forward and reverse primers for mouse TnC were 5’- GCGGTAGAACAGTTGACAGAG-3’ and 5’- CCAGCTCCTTGGTGCTGAT-3’.

Forward and reverse primers for mouse CARP were 5’- TGGCGATCGTGGAGAAGTTAA-3’ and 5’- TGCGTTTCCTCCACGACAT-3’.
